# Neuropsychological Assessment of a New Computerized Cognitive Task that Was Developed to Train Several Cognitive Functions Simultaneously

**DOI:** 10.3389/fpsyg.2016.00497

**Published:** 2016-04-12

**Authors:** Satoe Ichihara-Takeda, Kazuyoshi Takeda, Nozomu Ikeda, Kiyoji Matsuyama, Shintaro Funahashi

**Affiliations:** ^1^Department of Occupational Therapy, School of Health Science, Sapporo Medical UniversitySapporo, Japan; ^2^Department of Psychiatry, National Center of Neurology and Psychiatry, National Center HospitalTokyo, Japan; ^3^Kokoro Research Center, Kyoto UniversityKyoto, Japan

**Keywords:** cognitive training method, multiple functions, working memory, daily activity, human, neuropsychological test batteries

## Abstract

Recent studies have indicated that computerized cognitive training is effective as therapy for reducing the cognitive decline with aging and the dysfunction associated with neuropsychiatric illness. Although cognitive trainings that targets a specific function and multi-domain cognitive training have both been shown to have significant effects, we need one simple behavioral training paradigm to improve multiple domains of cognitive functions easily and simultaneously. We had developed a new computerized task that seeks to engage the cognitive functions of planning, mental calculation, and divergent thinking based on a working memory task in a single task. The purpose of this study was to assess the cognitive features of our new task by comparing the scores of seven known neuropsychological batteries in healthy elderly subjects. The relationships between performance in our task and the scores obtained by the neuropsychological batteries were examined. The percentage of correct performance on our task was correlated with the scores on the category fluency test, the digit span backward task, and the Trail making test B. Stepwise multiple regression analyses revealed that the scores on the category fluency test and the Trail making test B showed significant positive correlations with the percentage of correct performance on our task. Although the present study did not show high correlations between the percentage of correct performance on our task and working memory functions as a primary target, we observed mid-level correlations between the percentage of correct performance on our task and functions for divided attention and word fluency. Our new task requires not only working memory, but also attention and divergent thinking. Thus, this task might be a useful tool for training multiple cognitive functions simultaneously.

## Introduction

In our daily life, we often encounter situations that require various cognitive functions simultaneously, such as thinking, planning, and decision-making. These cognitive functions have been known to deteriorate with aging, as well as with various diseases, such as dementia, schizophrenia, attention-deficit hyperactivity disorder, and stroke. Computerized cognitive trainings have been shown to be effective for preventing such deterioration and improving existing dysfunction in the therapeutic treatment of patients and for improving cognitive ability in healthy subjects (Vinogradov et al., 2012; Lampit et al., 2014b). As a type of domain-specific cognitive training that targets a specific function, working memory training has been known to improve working memory capacity, attention ability, and fluid intelligence (Klingberg et al., 2005; Westerberg et al., 2007; Jaeggi et al., 2008; Au et al., 2016). It has even been suggested that the effects of this training can transfer to better performance of untrained tasks and untrained functions (Brehmer et al., 2012; Heinzel et al., 2014; Salminen et al., 2015). Attention training such as dual-task training method is known to be useful for improving an ability of divided attention (Lussier et al., 2012). Speed of processing training is known to be effective for improving everyday performance (Edwards et al., 2013). On the other hand, multi-domain cognitive training that includes some various cognitive tasks also showed positive effects. For example, the intervention based on the Cogpak package improved memory functions and information processing speed (Lampit et al., 2014a). The intervention of CogniFit improved working memory and executive function (Shatil et al., 2014). A house program improved inhibition and reasoning (van Muijden et al., 2012). And a cooking program improved executive control processing (Wang et al., 2011).

Although both the domain-specific or multi-domain cognitive trainings have been shown to exhibit positive effects in various cognitive functions, we often encounter situations that require various cognitive functions simultaneously in order to solve problems. To overcome our cognitive problem facing in our daily life, we needed to develop a cognitive task that improves different cognitive functions simultaneously. Therefore, we developed a new computerized cognitive task. The targeted cognitive functions in our task should be the functions that the dorsolateral prefrontal cortex (DLPFC) participates in. The DLPFC is an important brain area for understanding such important function as thinking, appropriate planning, judgment, decision-making, reasoning, and divergent thinking (Stuss and Benson, 1986; Goldman-Rakic, 1987; Fuster, 1997). Working memory is considered to be a key function for understanding how these cognitive functions are operated in the DLPFC (Baddeley, 1986; Goldman-Rakic, 1987). The delayed response task has been widely used as a typical working memory task to examine neural mechanisms of working memory in the DLPFC (Ichihara-Takeda and Funahashi, 2007). Therefore, our present task is developed based on the concept of the delayed response task, which is a short-time retention of several different information. We included the components of planning, mental calculation, and divergent thinking, which are also important functions of the DLPFC. The executive function, which is considered to be the coordinated operation of various processes to accomplish a particular goal in a flexible manner, is also an important function of the DLPFC (Funahashi, 2001). Since we intended to promote the coordinated operation of various cognitive functions to accomplish a particular goal naturally, we simulated activities and situations that could be found in daily life such as shopping or food preparation, on a computer. In the present study, we evaluated whether our new task is related to the intended cognitive functions. We examined what correlations were observed between the scores obtained from seven well-known neuropsychological batteries and the performance of our new task.

## Materials and methods

### Subjects

The participants of this study were 47 healthy elderly subjects (23 males and 24 females; age = 60–78 years; mean age = 69.3 ± 5.4 years; mean education = 13.0 ± 2.4 years). Inclusion criteria were: (i) Mini-Mental State Examination score of 23 or better; (ii) no history of head injury, cranial nerve disease, or serious medical disease. Fifty participants were recruited through a coordinator in a local community center. Three participants were excluded due to a low score on the Mini-Mental State Examination.

Written informed consent was obtained from each subject after the detailed explanation of the aim and procedure of the study. This experiment was approved by the ethics committee of Sapporo Medical University.

### New computerized cognitive training paradigm

**Figure 1:**
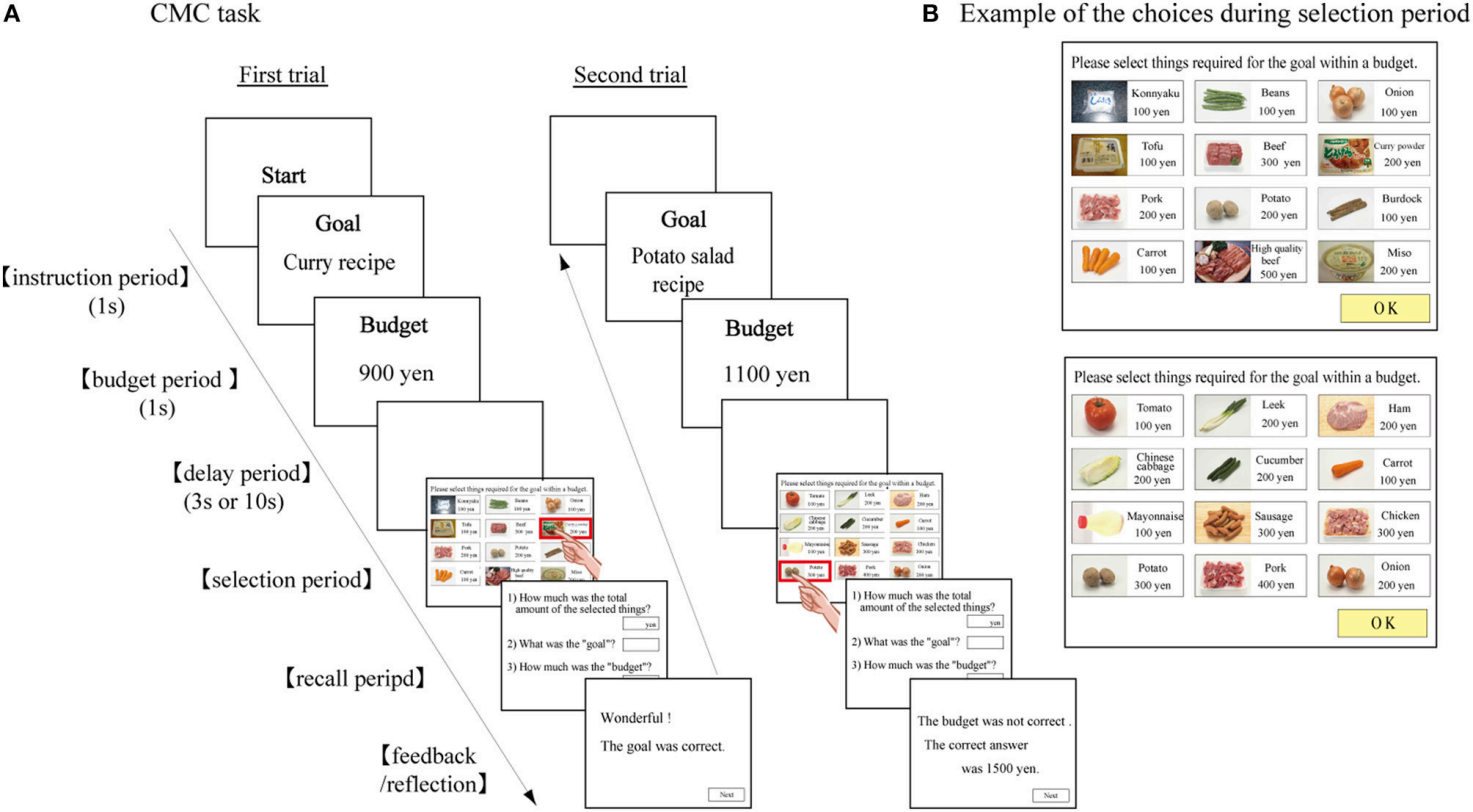
**Schematic diagram of CMC task. (A)** Temporal sequence of the task events in the CMC task. At the beginning of the trial, a “Start” signal is displayed for 1 s. The goal is then presented for 1 s (instruction period), followed by the presentation of the budget for 1 s (budget period). After a delay period of 3 or 10 s, 12 items are presented for selection. The subject is required to select necessary items to accomplish the goal within the budget, by touching the item on the computer screen, at which point the outer frame of the selected item turns red for 1 s. The subject needs to remember the selected items and their price. After the subject selects all of the necessary items, the subject is asked to specify the goal and the budget (recall period). The subject then receives feedback indicating whether or not their answers were correct. After an inter-trial interval, the next trial starts. **(B)** Two examples of the list of items presented during the selection period; top, “Curry recipe”; bottom, “Potato salad recipe.”

We developed a new computerized multiple cognitive task (CMC task) that developed for training several DLPFC functions simultaneously. In our task, subjects are asked to select necessary items from a list to achieve a goal without exceeding a pre-determined budget. A laptop computer with a touch-screen monitor (tc4400, HP, Japan) was used for the CMC task. Necessary information and stimuli were presented on the computer monitor. Figure 1A shows a schematic drawing of the CMC task. A trial started at the presentation of the start signal (the word “Start”) on the monitor for 1 s. Next, the goal that the subject needed to achieve (e.g., purchase the ingredients for a curry recipe) was presented on the monitor for 1 s (instruction period). Next, the budget that the subject could spend on the necessary items (e.g., 900 yen) was presented on the monitor for 1 s (budget period). A 3 or 10 s delay period was then introduced. After the delay period, a list of 12 items was presented along with their prices on the monitor (Figure 1B). The subject could then select the items needed to achieve the goal by touching the items on the monitor (selection period). Since the list included both necessary and unnecessary items, the subject needed to select necessary items (e.g., curry powder) and avoid selecting inappropriate items (e.g., miso). The subject needed to plan to purchase necessary items and select those items based on their personal knowledge, ideas, or preferences. For example, if the subject wanted to prepare beef curry, he/she could choose beef and some vegetables, and not choose pork. When the subject selected an item from the list, the background color of that item turned to red for 1 s. Therefore, the subject had to not only memorize the selected items and their prices during the selection period, but also calculate the running total cost of the selected items, since the budget was predetermined and limited. After the subject selected the necessary items, they were asked two questions (recall period): (1) “What was the goal?” and (2) “How much was the budget?” The subject needed to type the correct goal and budget. After the recall period, the subject received feedback from the computer: for example, “The goal was correct,” or “The amount of the budget was not correct. The correct amount was 1500 yen.” The next trial started after a 5 s interval.

In the CMC task, the experimenter can easily compose the goal, the budget, and the items available for selection based on the subject's interests and preferences. There are no limits to the task contents. In addition, the difficulty can be adjusted to match the subject's ability by changing the length of the delay period and the price of each item. However, in the present study, to compare the performance of our task across subjects, all subjects were tested under the same task conditions, so that they had the same goals, the same budget (700–1500 yen), the same length of the delay period (3 and 10 s), and the same range of item price (50–650 yen). The goals were familiar and popular Japanese home-style cooking recipes (Fujita, 2012; Kawakami, 2012), such as curry, potato salad, tempura, miso soup, hamburger, and so on. All subjects practiced once, which is different from the contents in the main session. Then, they performed 12 trials as the main session.

### Neuropsychological batteries

The subjects were tested using neuropsychological batteries to examine their abilities at general cognition, working memory, attention, set-shifting, and reasoning. This procedure took ~50 min for each subject. Each subject performed the following seven neuropsychological batteries: (i) The Mini-Mental State Examination (MMSE) measured each subject's general cognitive ability (Folstein et al., 1975). (ii) Category- and letter-fluency tests were used to measure the subject's verbal fluency. Each participant was asked to generate as many words that started with the same letter (letter fluency) or names of animals (category fluency) as possible within 1 min (Benton, 1968; Nemoto et al., 2007). (iii) The frontal assessment battery (FAB) measured frontal functions including mental flexibility, motor programming, sensitivity to interference, inhibitory control, and environmental autonomy (Dubois et al., 2000). (iv) The digit span task from the Wechsler Memory Scale-Revised (WMS-R) measured the subject's capacity for auditory working memory (Wechsler, 1987; Lezak et al., 2012). Performance of the digit span task depends on executive functions including working memory, cognitive regulation, and manipulation (Doi et al., 2013). (v) The visual memory span task from WMS-R measured visuo-spatial working memory capacity (Wechsler, 1987). (vi) Trail making test Parts A and B (TMT-A and TMT-B) measured the subject's abilities regarding selective attention (TMT-A) and divided alternative attention between sets of stimuli (TMT-B), respectively (Mitrushina et al., 1999; Lezak et al., 2012). (vii) Raven's progressive matrices measured the subject's non-verbal reasoning ability (Raven, 1995). These neuropsychological batteries were administered in the order shown.

### Statistical analysis

As the subject's behavioral scores in the CMC task, we calculated the percentages of correct recall of the goal and the budget. In addition, since the item lists used in the CMC task included inappropriate items for achieving the goal, we also calculated the number of inappropriate selections for each subject.

To determine the neuropsychological features of the CMC task, the relationships between the behavioral scores on the CMC task and the scores on the neuropsychological batteries were compared using Pearson's correlation coefficient. A stepwise multiple regression analysis was also used to examine whether potential predictors of neuropsychological batteries were independently associated with the parameters of the CMC task (the goal, budget, and number of inappropriate selections). Each variable of the neuropsychological batteries that exhibited a statistically significant correlation with a parameter of the CMC task was regarded as an independent variable. The parameters of the CMC task were considered as dependent variables. PASW Statistics version 18.0 (SPSS, Chicago, IL, USA) was used for this analysis, and the level of significance was set at 0.05.

## Results

### Behavioral performances of the CMC task and neuropsychological batteries

In the present study, we used the percentages of correct recall of the goal and the budget as behavioral scores for the CMC task. The number of inappropriate selections in the CMC task was also calculated. The average percentages of correct recall of the goal and the budget were 94.5% (SD = 9.7) and 71.6% (SD = 14.7), respectively. This difference was statistically significant (paired *t*-test, *t* = 11.5, *P* < 0.001). Thus, these results indicated it was more difficult to recall the budget than it was to recall the goal. The average number of inappropriate selections was 1.7 (SD = 1.6) items/trial.

Table 1 summarizes the scores of the neuropsychological batteries used for the present study in the 47 elder subjects. The mean scores for all these batteries were within the averages of normal elderly subjects published before (Kennedy, 1981; Wechsler, 1987; Raven, 1995; Nouchi et al., 2012; Doi et al., 2013). Thus, all subjects participated in the present study had normal ranges of abilities for examined cognitive functions.

**Table 1:** **Clinical characteristics of the participants**.

	**Mean (SD)**
Age (years) (range)	69.3 (5.4) 60–78
Education (years) (range)	13.0 (2.5) 6–16
MMSE (score)	28.2 (1.9)
FAB (score)	16.6 (1.9)
Letter fluency test (number/second)	10.3 (3.7)
Category fluency test (number/second)	15.9 (3.6)
Digit span forward (score)	7.1 (1.8)
Digit span backward (score)	5.6 (1.4)
Visual memory span forward (score)	7.7 (1.8)
Visual memory span backward (score)	6.6 (1.3)
Trail making test Part A (seconds)	42.6 (12.5)
Trail making test Part B (seconds)	98.1 (33.7)
Raven's progressive matrices (score)	29.3 (4.0)

*Values are presented as the mean (standard deviation). MMSE, Mini-Mental State Examination; FAB, Frontal Assessment Battery*.

### Relationships between behavioral scores on the CMC task and scores on neuropsychological batteries

To assess the validity of the CMC task for evaluating different cognitive functions, Pearson's correlation coefficients were used for examining relations between the behavioral scores on the CMC task and scores obtained from neuropsychological batteries (Table 2). The percentage of correct recall of the goal on the CMC task had significant positive correlations with the scores on the MMSE (*r* = 0.34, *p* < 0.05), the FAB (*r* = 0.29, *p* < 0.05), the category fluency test (*r* = 0.33, *p* < 0.05), and the digit span backward test (*r* = 0.33, *p* < 0.05), and significant negative correlations with the TMT-B (*r* = −0.45, *p* < 0.01; Table 2).

**Table 2:** **Correlations between performance on the CMC test and the scores on neuropsychological batteries**.

	**Recall of the goal**		**Recall of the budget**		**Number of inappropriate selections of items**	
	***r***	***p***	***r***	***p***	***r***	***p***
MMSE Total	0.34	< 0.05	0.24	n.s	−0.33	< 0.05
FAB	0.29	< 0.05	0.24	n.s	−0.20	n.s
Letter fluency test	0.24	n.s	0.25	n.s	−0.29	n.s
Category fluency test	0.33	< 0.05	0.50	< 0.001	−0.36	< 0.05
Digit span forward	0.26	n.s	0.16	n.s	−0.27	n.s
Digit span backward	0.33	< 0.05	0.29	< 0.05	−0.11	n.s
Visual memory span forward	0.13	n.s	0.11	n.s	0.12	n.s
Visual memory span backward	0.13	n.s	−0.04	n.s	0.13	n.s
Trail making test Part A	−0.22	n.s	−0.21	n.s	−0.01	n.s
Trail making test Part B	−0.45	< 0.05	−0.30	< 0.05	0.17	n.s
Raven's progressive matrices	0.26	n.s	0.21	n.s	−0.27	n.s

*MMSE, Mini-Mental State Examination; FAB, Frontal Assessment Battery. n.s., not significant*.

The percentage of correct recall of the budget on the CMC task had significant positive correlations with the scores on the category fluency test (*r* = 0.50, *p* < 0.001) and the digit span backward test (*r* = 0.29, *p* < 0.05), and significant negative correlations with the TMT- B (*r* = −0.30, *p* < 0.05; Table 2).

The numbers of inappropriate selections of items on the CMC task had significant negative correlations with the scores on the MMSE (*r* = −0.33, *p* < 0.05) and the category fluency test (*r* = −0.36, *p* < 0.05; Table 2).

Table 3 shows the neuropsychological batteries that were significantly related to the contents in the CMC task by a stepwise multiple regression analysis. Since the correlations between independent variables were not strong (|r| < 0.90), a stepwise multiple regression analysis was performed. The factor that was retained in the model for the recall of goal was the TMT-B (β = −0.45, *P* = 0.001). The factor that was retained in the model for the recall of budget was the category fluency test (β = 0.50, *P* < 0.001). The factor that was retained in the model for the number of inappropriate selections was the category fluency test (β = −0.36, *P* = 0.01).

**Table 3:** **Factors associated with the variables of the CMC task in a stepwise multiple regression analysis**.

**Dependent variable**	**Independent variable**	**β**	***p***
Recall of the goal	Trail making test Part B	−0.45	0.001
Recall of the budget	Category fluency test	0.50	< 0.001
Inappropriate selection	Category fluency test	−0.36	0.01

## Discussion

Recent studies have indicated that computerized cognitive training is effective as therapy for reducing the cognitive decline with aging and the dysfunction associated with neuropsychiatric illness. Although training that targets a specific function and multi-domain cognitive training have both been shown to have various significant effects (Vinogradov et al., 2012; Lampit et al., 2014b), we need one simple behavioral training paradigm to improve multiple domains of cognitive functions easily and simultaneously. We developed the CMC task, which was intended to be based on a working memory task and to engage several DLPFC functions such as planning, mental calculation, and divergent thinking for the accomplishment of a particular goal in a flexible manner, all in the same trial. In the present study, we evaluated our new task by comparing the scores to those of well-known neuropsychological batteries.

The present study showed that the subject's ability to correctly recall both the goal and the budget in the CMC task is significantly correlated with the score on the category fluency test, the digit span backward test, and the TMT-B. Stepwise multiple regression analyses revealed that performance on the TMT-B had a significant negative correlation with the percentage of correct recall of the goal, and that performance on the category fluency test had a significant positive correlation with the percentage of correct recall of the budget.

The percentages of correct recall of the goal and the budget in the CMC task were significantly correlated with the scores on the digit span backward test and the TMT-B. Performance of the digit span task depends on the capacity of working memory, cognitive regulation and manipulation, all of which are components of executive function (Doi et al., 2013). Performance of the TMT-B requires divided and alternative attention between a set of stimuli (Mitrushina et al., 1999; Lezak et al., 2012). Sánchez-Cubillo et al. (2009) indicated that performance of the TMT-B requires primarily working memory ability and secondarily task-switching ability. These results suggest that the CMC task may access the capacity of working memory, cognitive manipulation, divided attention, and task-switching.

The present study also revealed positive correlations between the percentages of correct recall of the goal and the budget in the CMC task and the scores on the category fluency test. The number of inappropriate selections was also correlated with the scores on the category fluency test. Previous studies have indicated that verbal fluency is an important factor for social function in schizophrenia patients (Nemoto et al., 2007) and that verbal fluency is an important predictor of functional outcomes in both schizophrenia and Alzheimer patients (Monsch et al., 1992; Green et al., 2000). Although verbal fluency has been considered to be an important functional predictor of mental disease, no previous report has demonstrated that working memory training provided a clear improvement (Morrison and Chein, 2011). An important feature of the CMC task is that it uses concrete activities and situations that are encountered in everyday life. The subject needs to freely select and arrange appropriate items among a variety of items using only their own memory and experience during the selection period in the CMC task. This feature may contribute to why the CMC task is related to category fluency.

The percentage of correct recall of the goal in the CMC task showed a significant positive correlation with the score on the MMSE. In the CMC task, the subject needs to maintain information regarding the correct goal and the correct budget during both the delay period and the selection period. Therefore, the significant positive, albeit weak, correlation between the percentage of correct recall of the goal and the score for the MMSE suggests that the CMC task may engage general cognitive functions.

The present results indicate that the cognitive requirements to correctly perform the CMC task are the functions for working memory, cognitive manipulation, divided attention, and word fluency. Although the present study did not show high correlations between the percentage of correct performance on our task and working memory functions as a primary target, we observed mid-level correlations between the percentage of correct performance on our task and functions for divided attention and word fluency. Our new task requires not only working memory, but also attention and divergent thinking. Thus, this task might be one of appropriate tools for training multiple cognitive functions simultaneously. We now could clarify cognitive features required in the CMC task. Therefore, we can next use the CMC task for cognitive training to elder persons with cognitive decline or patients with psychiatric disease and determine the effects of training using the CMC task on general cognitive capacities, the ability to apply these capacities in daily life, and motivation to do some activities.

## Author contributions

SI, KT, and SF contributed to the development of the task. SI, NI, and KM contributed to the evaluation of the task.

### Conflict of interest statement

The authors declare that the research was conducted in the absence of any commercial or financial relationships that could be construed as a potential conflict of interest.
